# Evaluating complete response prediction rates in locally advanced rectal cancer with different radiomics segmentation approaches

**DOI:** 10.3389/pore.2024.1611744

**Published:** 2024-04-17

**Authors:** Gizem Kaval, Merve Gulbiz Dagoglu Kartal, Sena Azamat, Eda Cingoz, Gokhan Ertas, Sule Karaman, Basak Kurtuldu, Metin Keskin, Neslihan Berker, Senem Karabulut, Ethem Nezih Oral, Nergiz Dagoglu Sakin

**Affiliations:** ^1^ Department of Radiation Oncology, Istanbul Faculty of Medicine, Istanbul University, Istanbul, Türkiye; ^2^ Department of Radiology, Istanbul Faculty of Medicine, Istanbul University, Istanbul, Türkiye; ^3^ Department of Radiology, Cam and Sakura City Hospital, Istanbul, Türkiye; ^4^ Department of Radiology, Bagcilar Training and Research Hospital, Istanbul, Türkiye; ^5^ Department of Biomedical Engineering, Yeditepe University, Istanbul, Türkiye; ^6^ Department of Emergency, Hackalibaba Hospital, Trabzon, Türkiye; ^7^ Department of General Surgery, Istanbul Faculty of Medicine, Istanbul University, Istanbul, Türkiye; ^8^ Department of Pathology, Istanbul Faculty of Medicine, Istanbul University, Istanbul, Türkiye; ^9^ Department of Medical Oncology, Istanbul Faculty of Medicine, Istanbul University, Istanbul, Türkiye

**Keywords:** rectal cancer, watch and wait, radiomics, radiotherapy, pathological complete response ratio

## Abstract

**Purpose:**

Studies examining prediction of complete response (CR) in locally advanced rectum cancer (LARC) from pre/post chemoradiotherapy (CRT) magnetic resonance imaging (MRI) are performed mostly with segmentations of the tumor, whereas only in two studies segmentation included tumor and mesorectum. Additionally, pelvic extramesorectal region, which is included in the clinical target volume (CTV) of radiotherapy, may contain information. Therefore, we aimed to compare predictive rates of radiomics analysis with features extracted from segmentations of tumor, tumor+mesorectum, and CTV.

**Methods and materials:**

Ninety-three LARC patients who underwent CRT in our institution between 2012 and 2019 were retrospectively scanned. Patients were divided into CR and non-CR groups. Tumor, tumor+mesorectum and CTV were segmented on T2 preCRT MRI images. Extracted features were compared for best area under the curve (AUC) of CR prediction with 15 machine-learning models.

**Results:**

CR was observed in 25 patients (26.8%), of whom 13 had pathological, and 12 had clinical complete response. For tumor, tumor+mesorectum and CTV segmentations, the best AUC were 0.84, 0.81, 0.77 in the training set and 0.85, 0.83 and 0.72 in the test set, respectively; sensitivity and specificity for the test set were 76%, 90%, 76% and 71%, 67% and 62%, respectively.

**Conclusion:**

Although the highest AUC result is obtained from the tumor segmentation, the highest accuracy and sensitivity are detected with tumor+mesorectum segmentation and these findings align with previous studies, suggesting that the mesorectum contains valuable insights for CR. The lowest result is obtained with CTV segmentation. More studies with mesorectum and pelvic nodal regions included in segmentation are needed.

## Introduction

Colorectal cancer is the third most common malignancy among all [1]. Recently, the recommended treatment of locally advanced rectum cancer (LARC) is “total neoadjuvant therapy,” which consists of long-term chemoradiotherapy (CRT) or short-term radiotherapy (RT) and consolidation or induction chemotherapy in suitable patients, followed by total mesorectal excision (TME) or “watch-and-wait” [2–4]. Pathological complete response (pCR) is observed at a rate of 15%–30% and it is important to truly detect these patients before surgery, who may be candidates for “watch-and-wait” [5].

Endoscopy and imaging are the most common methods for assessing treatment response. True prediction of clinical complete response (cCR) by endoscopy is approximately 85%, yet it does not discern extraluminal lesions [6]. The most suitable imaging method is magnetic resonance imaging (MRI) as it does not emit ionizing radiation and shows soft tissue with high resonance. If fibrosis develops inside the tumor after CRT, the viable tumor tissue under the fibrotic scar may not be detected with MRI [7, 8]. Diverse treatment responses necessitate an individualized treatment approach [9]. It is critical to develop markers that will predict unresponsive or more sensitive patients. Tumor heterogeneity, recognized as a potential mechanism contributing to resistance to CRT, arises from the phenomenon wherein neoplastic cells, stemming from a common progenitor undergo various mutations. This process leads to the emergence of distinct subpopulations within the tumor [10]. Furthermore, tumor cells may have molecular interactions with adipocytes in the peritumoral tissue and mesorectum, which support tumor progression and metastasis [11, 12]. Alongside their limitations, current diagnostic methods lack the capacity to comprehensively assess both intratumoral heterogeneity and peritumoral interactions that influence tumor behavior.

Radiomics is an innovative method developed with the aim of providing additional information that may affect the clinical approach derived from the reflections of the underlying pathophysiological features and intratumoral heterogeneity from medical images [13].

Radiomics studies in LARC have emphasized the noninvasive prediction of patients with complete response (CR) through preCRT and/or postCRT MRI [14] scans. Radiomics analysis was performed by segmenting only the tumor region in most of these studies. A study conducted by Shaish et al. in 2020, where they compared radiomics analyzes with different segmentations, is the first study to examine the predictive rate of pCR analysis with mesorectum segmentation. Segmented regions were tumor-only, mesorectum-only and tumor+mesorectum. The highest result was obtained from the radiomics analysis of mesorectum-only segmentation for pCR evaluation. In another study conducted in 2022, radiomics analysis from mesorectal fatty tissue segmentation was examined [15, 16]. Based on these studies, analysis of mesorectal tissue seems to be informative for pCR. In addition to the mesorectum, the pelvic extramesorectal region, which is encompassed within the clinical target volume (CTV) of RT due to the occasional presence of nodal metastases may also provide valuable insights of disease behaviour. Regarding the issue, we aimed to compare CR predictive rates of radiomics analysis operated with features extracted from segmentations of tumor-only, tumor+mesorectum, and CTV.

## Methods

### Patients

In our study, patients who were referred to our institution with a histopathologically confirmed diagnosis of LARC between 2012 and 2019 were retrospectively screened. The stage was determined following endoscopic and radiological assessments. The study cohort comprised patients who underwent CRT and surgery afterwards or those who remained under surveillance without recurrence, adhering to the “watch-and-wait” approach. Patients lacking appropriate protocol images were excluded from the study. MRI images were retrieved from the Picture Archiving and Communication System.

Patients who attained a pathological stage of ypT0N0 were classified as pCR, while patients who met the criteria for the “watch-and-wait” strategy and remained recurrence-free for 4 years were categorized as cCR. In both instances, these patients were collectively evaluated as demonstrating CR.

### Neoadjuvant treatment and response evaluation

We conduct simulation computed tomography (SCT) scans with a cross-sectional interval of 0.3 cm for RT planning. CTV and gross tumor volume (GTV) are delineated following the RTOG contouring atlas [17].

RT is administered at a dose of 45–50.4 Gray (Gy) in 25–28 fractions. RT planning techniques were either three-dimensional conformal RT or intensity-modulated RT via Varian and Eclipse planning systems. Treatments are carried out with linear accelerator devices.

Simultaneously with RT, all patients were administered either capecitabine (825 mg/m^2^ orally in two daily doses, daily) or 5-fluorouracil (daily dose ranging from 200 to 225 mg/m^2^ by infusion) through a port.

Subsequent to neoadjuvant treatment, patients underwent evaluation via endoscopic techniques and MRI scans at 6–8 weeks. Surgical interventions were scheduled within a window of 6–10 weeks. Dworak Regression Score was employed to assess tumor regression [18]. Adjuvant chemotherapy was administered based on the pathology findings.

As for adjuvant therapy, 19 patients received 6–8 cycles of CAPOX (capecitabine+oxaliplatin), and 53 patients received 8–2 cycles of FOLFOX (5 Fluorouracil+oxaliplatin). Adjuvant chemotherapy was not administered to 20 patients. Notably, all patients enrolled in the “watch-and-wait” approach received at least 8 cycles of FOLFOX regimen.

Patients who met the specified criteria, as outlined in the trial conducted by Maas, were categorized as having achieved cCR [19].

### Image data acquisition and segmentation

MRI images were acquired utilizing 1.5 Tesla MRI devices, the Philips Achieva (Philips Medical Systems, Netherlands), and Symphoni (Siemens, Erlangen, Germany) models. The standard MRI imaging protocols for Turbo-spin-eco, T2-weighted sequences in the axial plane are as follows: TR/TE is 3,000/100 msec, image matrix of 348 × 278, a field of view measuring 210 × 228, and a cross-section thickness of 3 mm. Furthermore, the administration of iohexol contrast agent was performed at a dosage of 0.2 mL per kilogram during the MRI procedure.

**FIGURE 1:**
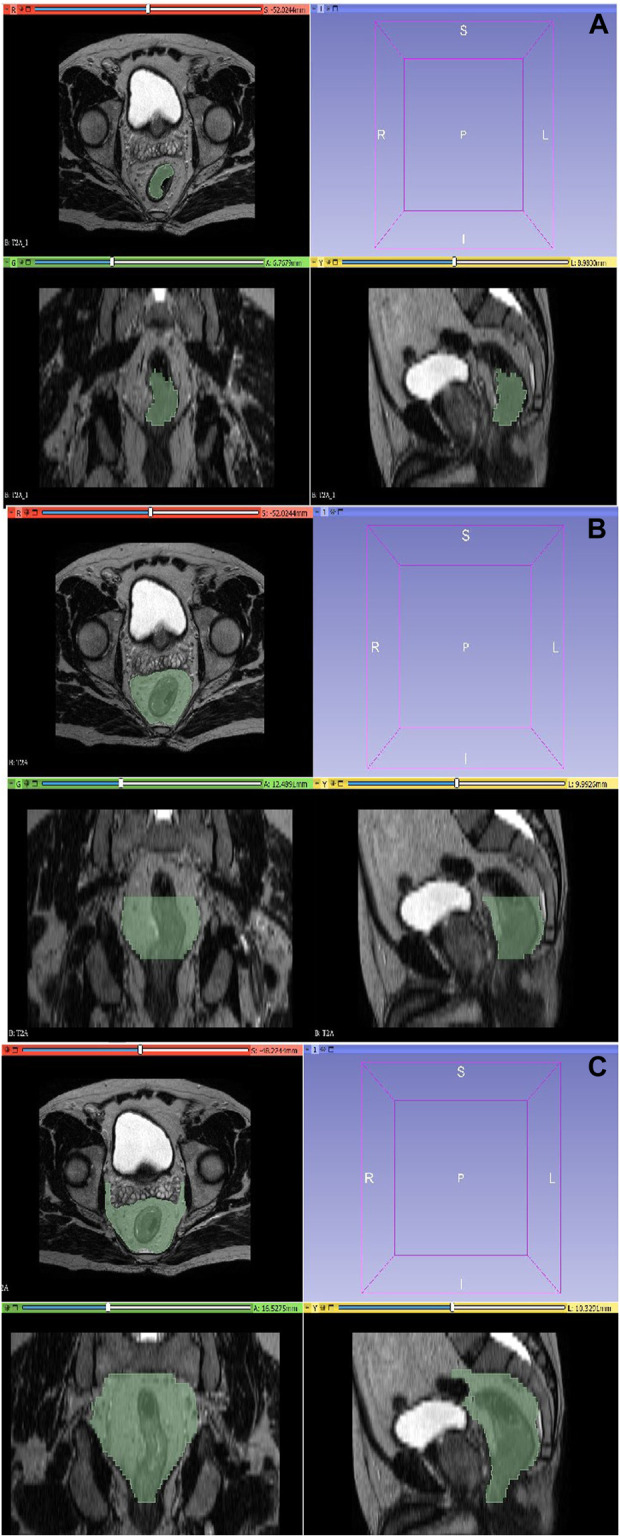
Segmentation with 3D-Slicer. **(A)** Tumor region. **(B)** Combined tumor-mesorectum. **(C)** CTV region.

A total of 93 contrast-enhanced abdominal preCRT MRI images from two MRI devices were anonymized, uploaded to MRIcron program in DICOM format, and converted to “nii.gz” format. Subsequently, the segmentation process was conducted through manual delineation using the 3D Slicer program. This collaborative effort involved radiation oncology (G.K.) and radiology (E.C.) residents with 6 years of experience and an associate professor radiologist (M.G.D.K.) with 16 (See Figure 1).

The segmentation of the tumor region was executed on T2 sequences, encompassing its complete volumetric extent while excluding vascular structures, calcifications, and air densities. This segmentation preserved the three-dimensional structural representation of the tumor as visualized in axial sections. Concurrently, the mesorectal region was delineated, incorporating the rectum and the adjacent mesorectal tissues along the craniocaudal extension of the tumor, without encompassing adjacent organs in the vicinity. The recommended CTV definition entails the inclusion of the entire mesorectum, perirectal area, presacral region, and the internal iliac lymph node regions in the axial plane and craniocaudally commences from the promontory and extends downward to a point 2 cm below the tumor. The delineation of the CTV was performed in a manner that excluded adjacent organs.

### Feature extraction and selection

The PyRadiomics 2.2.0, an extension of the open-source Python program for feature extraction,^1^ was employed in the study. Prior to analysis, N4 bias correction was applied to the data. The image discretization bin width was set at 5, voxel array shift at 300, normalization scale at 100, and resampling at 3 mm × 3 mm × 3 mm. The extracted features encompassed both first-order characteristics such as mean gray value level, entropy, standard deviation, skewness (a measure of asymmetry around the mean), and kurtosis (a measure of histogram flatness) and high-order features derived from gray-level co-occurrence, gray-level dependence, gray-level run-length and gray-level size zone matrixes, and wavelet and Laplacian of Gaussian filters.

The analysis continued within the Python 2.3 environment, utilizing Jupyter Notebook and the PyCaret Library. The data underwent Z-score normalization as a preprocessing step. A multicollinearity threshold of 0.9 and an outlier removing threshold of 0.05 were applied. The dataset was divided into training and test sets, adhering to a ratio of 0.7 for training and 0.3 for testing. Cross-validation was performed five times. For feature selection, the “classical” technique was chosen and applied.

### Machine learning

To address the imbalanced distribution within the dataset, the Synthetic Minority Over-sampling Technique (SMOTE) was employed. Subsequently, a Pearson correlation test was conducted, and correlation matrixes were obtained. For features from each three segmentations, machine-learning models were independently evaluated using the training dataset to predict the two groups. Subsequently, the method that yielded the most favorable AUC result was further assessed using the test dataset.

## Results

### Patient and treatment characteristics

Among 93 patients, 23 (25%) were female, and 70 (75%) were male. The median age at diagnosis was 58 years, with a median follow-up period of 70 months (8–125). Eighty-one patients underwent TME. Among these surgical cases, 64 patients underwent Low Anterior Resection, while 17 patients underwent Abdominoperineal Resection. pCR was observed in 13 of the surgically treated patients, accounting for 16% of this subgroup. Information regarding the patients’ initial stage as determined by MRI and colonoscopy, as well as other characteristics, is presented in Table 1. Furthermore, 12 patients were determined to be cCR based on the results of MRI, PET/CT, and colonoscopy examinations and were managed according to the “watch-and-wait” protocol. During their follow-up, these patients underwent digital rectal examinations every 3 months, colonoscopies and MRI assessments every 6 months. None of these patients exhibited recurrence over the course of the 4-year follow-up period. In summary, CR, encompassing both pCR and cCR categories, was observed in 26.88% of all patients.

**TABLE 1:** Patients characteristics.

	*n* = 93		%
Median Age	58 (26–80)		
Male	70		75
Female	23		25
Tumor Localisation			
Distal	41		44
Middle	42		45
Proximal	10		11
cT			
T2	7		7
T3a	23		25
T3b	23		25
T3c	24		26
T3d	1		1
T4a	6		6
T4b	9		10
cN			
N0	15		16
N1	26		28
N1c	4		4
N2	48		52
CRM			
Negative	62		66
Positive	31		34

CRM, circumferential resection margin; cT, clinical T stage; cN, clinical N stage.

### Feature selection and construction of the radiomics model

A total of 1,132 radiomics features were extracted from each three different segmentations. Following normalization, the SMOTE and Pearson correlation test was conducted. For each radiomics features derived from three different segmentations, 15 machine-learning models were evaluated for the best AUC result, respectively.

### Performances of radiomics models operated with features from different segmentations

The “Light Gradient Boosting Machine Classifier” demonstrated the highest AUC value (AUC:0.84) for the analysis of tumor region segmentation. For the analysis of tumor+mesorectum segmentation, the “Logistic Regression” model exhibited the highest AUC in the training set (AUC:0.81). Meanwhile, for analysis of the CTV segmentation, the “Gaussian Naïve Bayes” model yielded the highest AUC value (AUC: 0.77).

In the test set, the highest AUC values for these analyzes of these respective segmentations were as follows: 0.85 for the tumor segmentation, 0.83 for the tumor+mesorectum segmentation, and 0.72 for the CTV segmentation.

Sensitivity and specificity rates in the test set were measured as 76% and 71% for tumor segmentation, 90% and 67% for tumor+mesorectum segmentation, and 76% and 62% for CTV segmentation, respectively.

**FIGURE 2:**
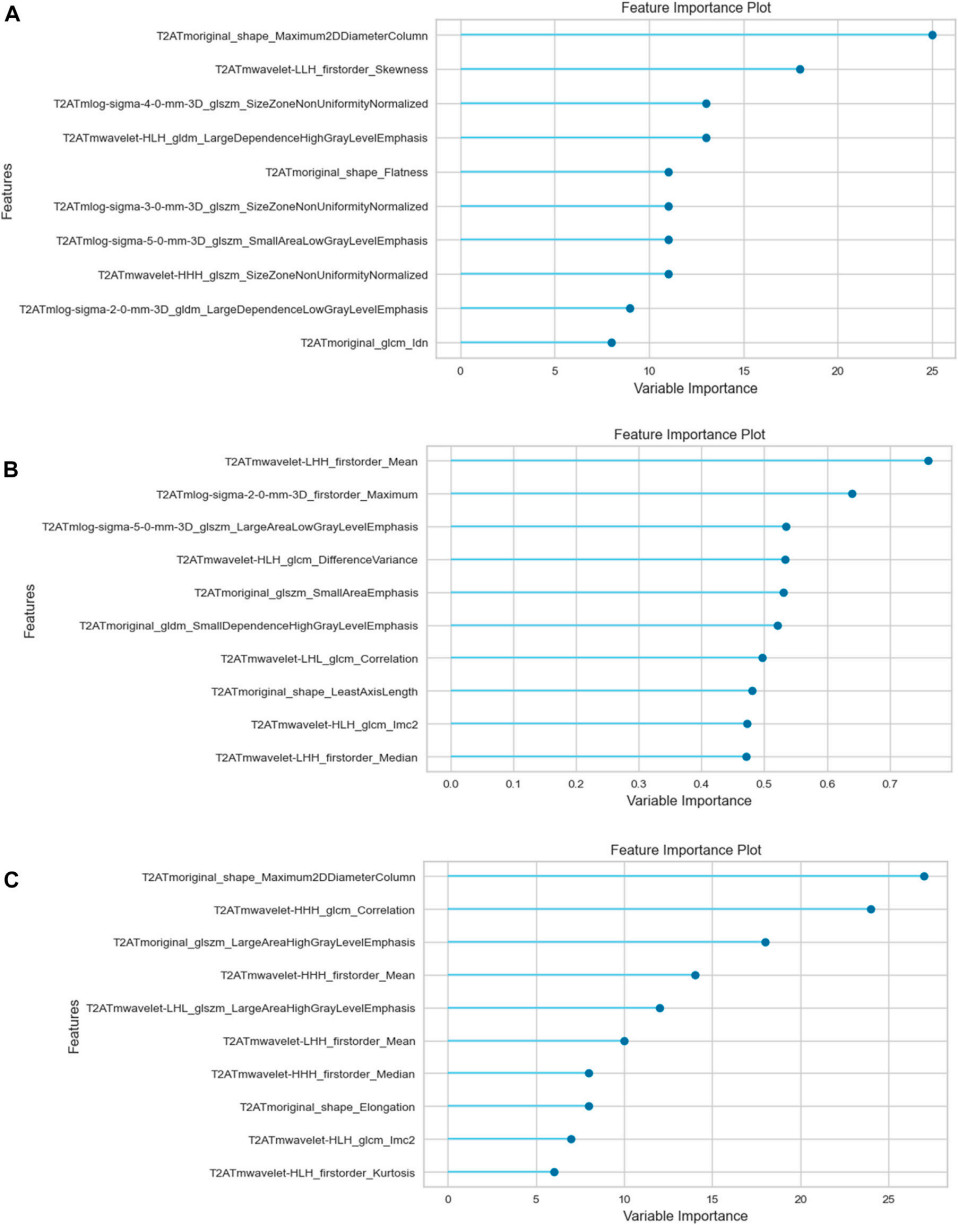
Feature importance plots. **(A)** Tumor. **(B)** Tumor+Mesorectum. **(C)** CTV.

**FIGURE 3:**
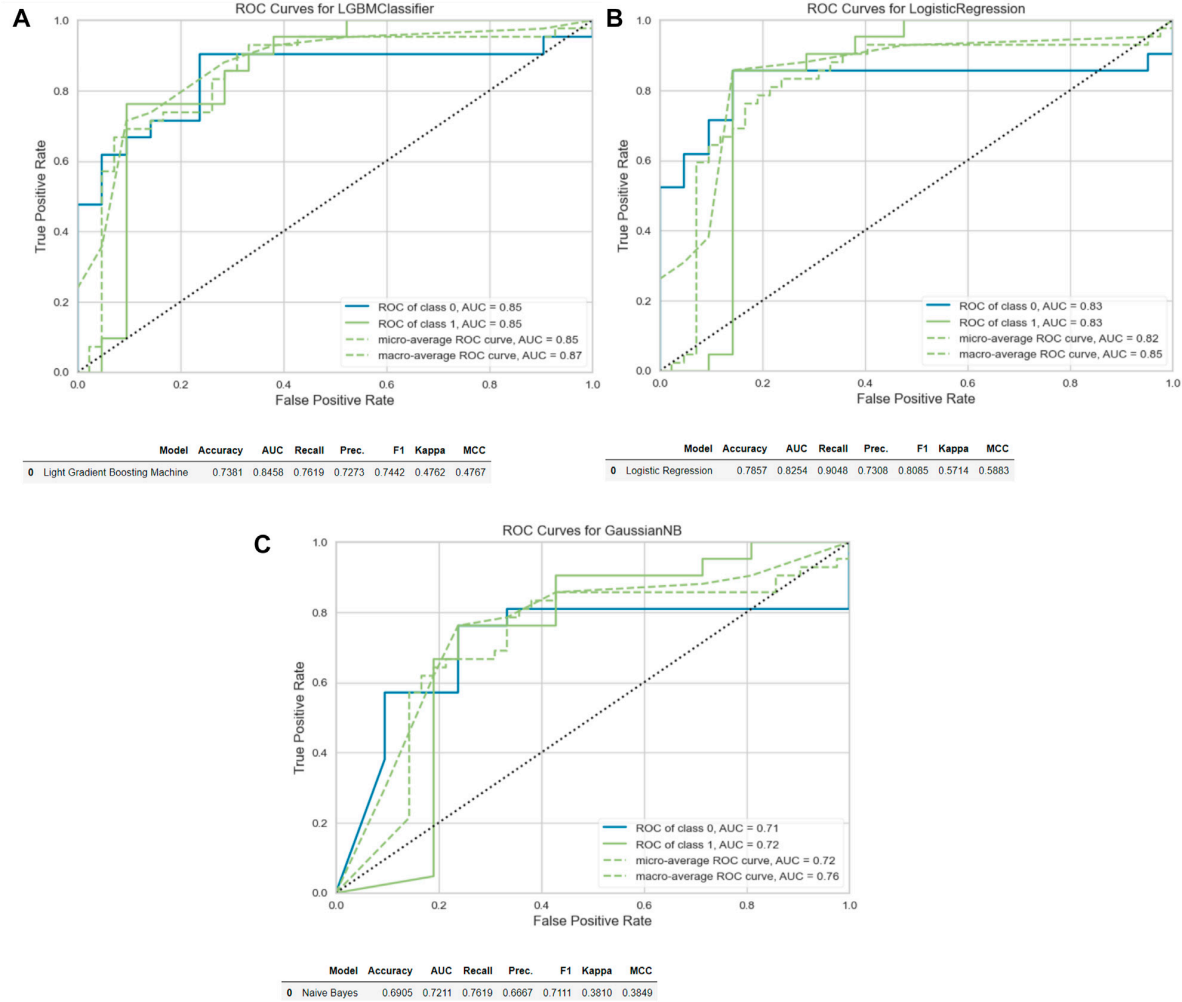
ROC curves and accuracy, AUC, recall and precision results. **(A)** Tumor. **(B)** Tumor+Mesorectum. **(C)** CTV.

Table 2 presents the calculated values, including AUC, sensitivity, specificity, positive odds ratio, negative odds ratio, positive predictive value (PPV), negative predictive value (NPV), and accuracy, obtained from each three analyzes with different segmentations. See Figures 2, 3 for selected features and ROC curves.

**TABLE 2:** AUC, sensitivity, specifity, positive odds ratio, negative odds ratio, positive and negative predictive values and accuracy results of the test set.

Segmentation Type	AUC	Sensitivity	Specificity	Positive Odds Ratio	Negative Odds Ratio	Positive Predictive Value	Negative Predictive Value	Accuracy
Tumor	0.85	76%	71%	2.67	0.33	73%	7%5	74%
Tumor + Mesorectum	0.83	90%	67%	2.71	0.14	73%	87,5%	79%
CTV	0.72	76%	62%	2.00	0.38	67%	72%	69%

AUC, area under curve; CTV, clinical target volume.

## Discussion

In our research, we focused on comparing radiomics analyzes for predicting CR based on preCRT MRI images derived from the segmentation of the tumor, tumor+mesorectum, and CTV regions in LARC patients who underwent neoadjuvant therapy. Our findings revealed that the highest AUC was achieved when analyzing solely the tumor segmentation.

The AUC values in the training set for analyzing three segmentations were as follows: 0.84 for tumor-only, 0.81 for tumor+mesorectum and 0.77 for CTV. In the test set, these AUC values were found to be 0.85 for tumor-only, 0.83 for tumor+mesorectum, and 0.72 for CTV segmentations, respectively. Regarding sensitivity, the test set yielded values of 76% for tumor-only, 90% for tumor+mesorectum, and 76% for CTV segmentations. In terms of specificity, the test set exhibited 71% for tumor-only, 67% for tumor+mesorectum, and 62% for CTV segmentations.

Radiomics for LARC has been evaluated in many studies. Models have been developed for the prediction of pCR, survival, and recurrence using preCRT and/or postCRT MRI images [14]. In the realm of rectal cancer, where locally advanced stages can still yield prolonged survival outcomes, there is a growing emphasis on personalized treatment strategies aimed at minimizing side effects. The ability to predict pCR based on preCRT MRI data has emerged as a valuable guiding marker for tailoring individualized treatment approaches.

Studies to date have mostly focused on pCR prediction through radiomics analysis of tumor region segmentations from preCRT MRI images. It is noteworthy that the behavior of rectal tumors is not solely dictated by the tumor itself; it is profoundly influenced by the transformation of peritumoral stromal tissue and the resultant alterations in the microenvironment [11, 12]. Consequently, the mesorectal tissue is deemed to harbor crucial insights into the disease. However, only two studies incorporated the mesorectum within the segmentation region [15, 16]. In 2020, Shaish et al. conducted a comparative analysis of radiomics, exploring various segmentation approaches encompassing tumor, mesorectum, and tumor+mesorectum regions. Their study reported that analysis from tumor+mesorectum segmentation achieved the highest AUC score (AUC: 0.80) for tumor regression scoring, underlining its significance. Subsequently, in 2022, Jayaprakasam et al. conducted analysis with mesorectal fatty tissue segmentation and achieved a AUC result of 0.89.

In datasets characterized by non-uniform distributions, such as the dataset in this study, the commonly employed parameter for assessing outcomes is the AUC. Consequently, AUC results were employed in our investigation for comparative analysis. It is important to acknowledge, however, that certain literature sources posit that metrics such as recall (sensitivity) and precision (PPV) may carry enhanced significance in certain contexts [20]. Accordingly, our study aimed to contextualize our findings by incorporating these metrics into our analysis.

Based on the AUC results, radiomics analysis of tumor segmentation exhibited the most favorable performance in both training and test datasets. Nevertheless, it is noteworthy that segmentation of tumor+mesorectum demonstrated superior accuracy, sensitivity, and PPV in the test, and superior sensitivity in the training set. This observation leads us to assert that the incorporation of mesorectal segmentation into future response assessment studies for LARC may harbor considerable potential.

In the context of the “watch-and-wait” protocol, an imperative prerequisite for patients to qualify as cCR is the identification of a negative lymph node on MRI [19]. Notably, while not encountered within our patient cohort, cases featuring ypT0N1 status have been reported in literature [21]. It is pertinent to emphasize that relying on radiomics based solely on tumor segmentation may prove insufficient in predicting CR. This aspect assumes significance as radiomics is being explored as a potential imaging-based biomarker guiding the selection of optimal neoadjuvant treatment regimens. To this end, a solitary study has undertaken radiomics analysis inclusive of lymph node segmentation on preCRT MRI scans. The outcomes of this study revealed an AUC of 0.91, underscoring the feasibility of accurately detecting a nodal CR from preCRT MRI [22] images.

Considering these findings, we posited that radiomics analysis with CTV region segmentation, encompassing both tumor and pelvic lymph node regions, could yield meaningful insights. However, the resultant AUC of 0.71 was found to be lower than that achieved through tumor-only and tumor+mesorectum segmentations. It is conceivable that, the presence of the tumor-free rectal region within the delineated CTV during segmentation, may have affected the obtained results negatively. In subsequent studies, it is advisable to consider a multi-sequence approach and to exclude the tumor-free rectum from the CTV region during the segmentation process.

Local recurrence rate after TME and neoadjuvant treatments generally falls within the range of 5%–10%, varying across different studies. A substantial proportion of such recurrences manifest within the extramesorectal lateral pelvic nodal region. The implementation of lateral pelvic nodal dissection remains a subject of controversy within the existing literature [23]. Consequently, while analysis with mesorectal segmentation has yielded the most favorable outcomes in terms of accuracy and sensitivity, there is a rationale to consider the assessment of the lateral pelvic nodal region within response evaluation studies with radiomics analysis, as it may offer valuable guidance for tailoring individualized treatment strategies.

It is important to note that, focus of most studies lies in the inclusion of patients who have attained a pCR, excluding patients with cCR. This practice carries the accidental risk of omitting a segment of patients who have indeed achieved a CR from the evaluative framework. In an effort to mitigate this potential limitation and maintain consistency, our study encompassed patients who exhibited cCR and have, to date, remained free from any relapse during the follow-up period.

Furthermore, it is worth highlighting that our study adopted a distinctive approach by not restricting the patient cohort to data acquired from a single MRI device, unlike most studies. Instead, we performed radiomics analysis using heterogeneous MRI data from two devices, resulting in an AUC value of 0.84.

Several limitations are worth acknowledging in our study. Firstly, its retrospective nature may be considered a potential limitation. Nonetheless, it is essential to emphasize that patient evaluations were conducted prospectively. Furthermore, the absence of an assessment regarding multiple readers and the lack of an interobserver reproducibility evaluation can be perceived as a limitation. Nevertheless, it is plausible that for volumes characterized by such standardized boundaries, such as CTV, the interreader variations are likely to be less pronounced compared to tumor segmentation. This aspect, however, could serve as a topic for future investigations. Moreover, it should be acknowledged that the models developed in our study were not subjected to evaluation using an external validation dataset. The primary rationale behind this decision lies in the objective of our study, which primarily aimed to compare outcomes across analyzes of different region segmentations rather than constructing a predictive model. Therefore, external validation was not prioritized for the specific research objectives of this study.

## Conclusion

In our research, we conducted a comparative analysis of radiomics assessments based on preCRT MRI images, focusing on three distinct segmentation areas: tumor-only, tumor combined with the mesorectum, and CTV. Although the highest AUC result was achieved with radiomics analysis of tumor segmentation, our investigation revealed that the highest accuracy and sensitivity were observed with radiomics analysis of combined tumor and mesorectum segmentations. Our findings align with previous studies, suggesting that the mesorectum contains valuable insights for the prediction of CR.

Finally, although the lowest predictive performance was observed with the analysis of CTV segmentation, predictive results of this region could be improved within further trials, considering the fact that lateral pelvic nodal region is also a possible local metastasis area and should be involved in CR prediction analyzes of LARC. There is a need for further research, encompassing larger patient cohorts and involving multiple readers with different segmentation methods to explore the inclusion of the mesorectum and pelvic nodal regions within the segmentation area for the prediction of CR as well as distant metastasis and prognosis in LARC.

## Data Availability

The raw data supporting the conclusion of this article will be made available by the authors, without undue reservation.

## Footnotes

1 Accessible at http:/radiomics.io/pyradiomics.html

## References

[1] GLOBOCAN. Estimated age-standardized incidence rates (World) in 2020, rectum. France: GLOBOCAN (2020). Available from: https://gco.iarc.fr/ (Accessed February 2023).

[2] Bahadoer RR Dijkstra EA van Etten B Marijnen CAM Putter H Kranenbarg EM Short-course radiotherapy followed by chemotherapy before total mesorectal excision (TME) versus preoperative chemoradiotherapy, TME, and optional adjuvant chemotherapy in locally advanced rectal cancer (RAPIDO): a randomised, open-label, phase 3 trial. Lancet Oncol (2021) 22:29–42. 10.1016/S1470-2045(20)30555-6 33301740

[3] Cisel B Pietrzak L Michalski W Wyrwicz L Rutkowski A Kosakowska E Long-course preoperative chemoradiation versus 5 × 5 Gy and consolidation chemotherapy for clinical T4 and fixed clinical T3 rectal cancer: long-term results of the randomized Polish II study. Ann Oncol (2019) 30:1298–303. 10.1093/annonc/mdz186 31192355

[4] Jin J Tang Y Hu C Jiang LM Jiang J Li N Multicenter, randomized, phase III trial of short-term radiotherapy plus chemotherapy versus long-term chemoradiotherapy in locally advanced rectal cancer (STELLAR). J Clin Oncol (2022) 40:1681–92. 10.1200/JCO.21.01667 35263150 PMC9113208

[5] Maas M Nelemans PJ Valentini V Das P Rödel C Kuo L-J Long-term outcome in patients with a pathological complete response after chemoradiation for rectal cancer: a pooled analysis of individual patient data. Lancet Oncol (2010) 11:835–44. 10.1016/S1470-2045(10)70172-8 20692872

[6] Maas M Lambregts DM Nelemans PJ Heijnen LA Martens MH Leijtens JW Assessment of clinical complete response after chemoradiation for rectal cancer with digital rectal examination, endoscopy, and MRI: selection for organ-saving treatment. Ann Surg Oncol (2015) 22:3873–80. 10.1245/s10434-015-4687-9 26198074 PMC4595525

[7] Lambregts DM Beets GL Maas M Curvo-Semedo L Kessels AG Thywissen T Tumour ADC measurements in rectal cancer: effect of ROI methods on ADC values and interobserver variability. Eur Radiol (2011) 21:2567–74. 10.1007/s00330-011-2220-5 21822946 PMC3217149

[8] Sassen S de Booij M Sosef M Berendsen R Lammering G Clarijs R Locally advanced rectal cancer: is diffusion weighted MRI helpful for the identification of complete responders (ypT0N0) after neoadjuvant chemoradiation therapy? Eur Radiol (2013) 23:3440–9. 10.1007/s00330-013-2956-1 23832319

[9] Dattani M Marijnen C Moran B Tait D Cunningham C Rodriguez-Bigas M Session 4: shaping radiotherapy for rectal cancer: should this be personalized? Colorectal Dis (2018) 20(Suppl. 1):92–6. 10.1111/codi.14087 29878670

[10] Hardiman KM Ulintz PJ Kuick RD Hovelson DH Gates CM Bhasi A Intra-tumor genetic heterogeneity in rectal cancer. Lab Invest (2016) 96:4–15. 10.1038/labinvest.2015.131 PMC4695247 26568296

[11] Chu K Bos SA Gill CM Torriani M Bredella MA. Brown adipose tissue and cancer progression. Skeletal Radiol (2020) 49:635–9. 10.1007/s00256-019-03322-w 31650208

[12] Conti G Calderan L Quintero Sierra LA Conti A Ossanna R Boschi F Tumor and peritumoral adipose tissue crosstalk: de-differentiated adipocytes influence spread of colon carcinoma cells. Tissue Cell (2023) 80:101990. 10.1016/j.tice.2022.101990 36542947

[13] Gillies RJ Kinahan PE Hricak H. Radiomics: images are more than pictures, they are data. Radiology (2016) 278:563–77. 10.1148/radiol.2015151169 26579733 PMC4734157

[14] Miranda J Horvat N Araujo-Filho JAB Albuquerque KS Charbel C Trindade BMC The role of radiomics in rectal cancer. J Gastrointest Cancer (2023) 54:1158–80. 10.1007/s12029-022-00909-w 37155130 PMC11301614

[15] Jayaprakasam VS Paroder V Gibbs P Bajwa R Gangai N Sosa RE MRI radiomics features of mesorectal fat can predict response to neoadjuvant chemoradiation therapy and tumor recurrence in patients with locally advanced rectal cancer. Eur Radiol (2022) 32:971–80. 10.1007/s00330-021-08144-w 34327580 PMC9018044

[16] Shaish H Aukerman A Vanguri R Spinelli A Armenta P Jambawalikar S Radiomics of MRI for pretreatment prediction of pathologic complete response, tumor regression grade, and neoadjuvant rectal score in patients with locally advanced rectal cancer undergoing neoadjuvant chemoradiation: an international multicenter study. Eur Radiol (2020) 30:6263–73. 10.1007/s00330-020-06968-6 32500192

[17] Myerson RJ Garofalo MC El Naqa I Abrams RA Apte A Bosch WR Elective clinical target volumes for conformal therapy in anorectal cancer: a radiation therapy oncology group consensus panel contouring atlas. Int J Radiat Oncol Biol Phys (2009) 74:824–30. 10.1016/j.ijrobp.2008.08.070 19117696 PMC2709288

[18] Dworak Lk O Hoffmann A. Pathological features of rectal cancer after preoperative radiochemotherapy. Int J Colorectal Dis (1997) 12:19–23. 10.1007/s003840050072 9112145

[19] Maas M Beets-Tan RG Lambregts DM Lammering G Nelemans PJ Engelen SM Wait-and-see policy for clinical complete responders after chemoradiation for rectal cancer. J Clin Oncol (2011) 29:4633–40. 10.1200/JCO.2011.37.7176 22067400

[20] Saito T Rehmsmeier M. The precision-recall plot is more informative than the ROC plot when evaluating binary classifiers on imbalanced datasets. PLoS One (2015) 10:e0118432. 10.1371/journal.pone.0118432 25738806 PMC4349800

[21] Kim SH Chang HJ Kim DY Park JW Baek JY Kim SY What is the ideal tumor regression grading system in rectal cancer patients after preoperative chemoradiotherapy? Cancer Res Treat (2016) 48:998–1009. 10.4143/crt.2015.254 26511803 PMC4946373

[22] Zhang S Tang B Yu M He L Zheng P Yan C Development and validation of a radiomics model based on lymph-node regression grading after neoadjuvant chemoradiotherapy in locally advanced rectal cancer. Int J Radiat Oncol Biol Phys (2023) 117:821–33. 10.1016/j.ijrobp.2023.05.027 37230433

[23] Otero de Pablos J Mayol J. Controversies in the management of lateral pelvic lymph nodes in patients with advanced rectal cancer: east or west? Front Surg (2019) 6:79. 10.3389/fsurg.2019.00079 32010707 PMC6979275

